# Malignant Fibrous Histiocytoma of the Kidney Treated with Nephrectomy and Adjuvant Radiotherapy: A Case Report

**DOI:** 10.1155/2010/802026

**Published:** 2010-09-28

**Authors:** Rita Marchese, Pantaleo Bufo, Giuseppe Carrieri, Giuseppe Bove

**Affiliations:** ^1^Department of Radiotherapy, Policlinico Ospedali Riuniti, University Hospital, 71100 Foggia, Italy; ^2^Department of Pathology, Policlinico Ospedali Riuniti, University Hospital, 71100 Foggia, Italy; ^3^Department of Urology, Policlinico Ospedali Riuniti, University Hospital, 71100 Foggia, Italy

## Abstract

Malignant fibrous histiocytoma (MFH) usually presents in the extremities or retroperitoneum. Cases involving the kidney are rare and portend a poor prognosis. Although radical nephrectomy is the most beneficial curative choice for this neoplasm, patients are often treated with adjuvant chemotherapy due to high risk of local recurrence and distant metastases. We describe a case of a 68-year-old woman affected by MFH, treated with both nephrectomy and radiotherapy without systemic therapy showing an unexpected twenty-four-month postsurgery survival outcome.

## 1. Introduction

Malignant fibrous histiocytoma (MFH) is the most common subtype of soft-tissue sarcoma in adults, and its characteristics were firstly described in 1964 by O'Brien and Stout [1]. MFH frequently localizes to the extremities and retroperitoneum, and rarely to the kidney [2–5]. Renal localization is characterized by high risk of local and distant recurrence, unfavorable prognosis, and complicated clinical and histopathological diagnosis, which is very often a diagnosis of exclusion. The role of adjuvant therapy for renal MFH is unclear, because of the insufficient number of cases offered. We describe here a case of primary renal MFH and the beneficial role of adjuvant radiotherapy treatment.

## 2. Case Report

**Figure 1:**
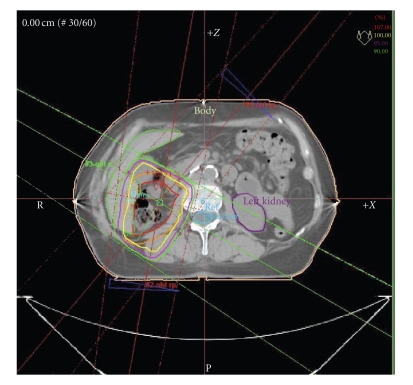
Computer tomography-based treatment plan with a combination of three fields (left anterior oblique, right posterior oblique, and right lateral oblique).

A 68-year-old woman with abdominal pain and low-grade fever of two-month duration was admitted to the hospital. Clinical examination was unremarkable and no palpable mass was found by physical inspection. Laboratory analysis revealed anemia (Hb 9.9 gm/dL) and leukocytosis (23000/*μ*L with normal range: 4000–11000/*μ*L). All the other biochemical data, including renal function and urine analysis, were normal. Chest X ray was unremarkable. Contrast-enhanced computed tomography (CT) showed a large mass of approximately 8 cm in diameter adjacent to the lower pole of the right kidney, which exhibited enhancement with contrast medium. The contralateral kidney and adjacent organs appeared normal and no enlarged lymph nodes were observed in the abdomen. A magnetic resonance imaging (MRI) was recommended but the patient refused the procedure because of severe claustrophobia. Based on the data collected, the preoperative diagnosis was of right renal cell carcinoma. Radical right nephrectomy was then performed by thoracoabdominal approach. Postoperative course was uneventful. The gross specimen included tissue from right kidney, ureter, right renal hilum and paracaval nodes. Nephrectomy specimen showed a pale yellow color, an uncircumcised solid lesion (maximum diameter 11 cm) confined to the lower pole of the right kidney, infiltrating the renal capsule and the perirenal adipose tissue. Multiple surgical margins were not involved. The tumor was histologically diagnosed as fibroblastic-pleomorphic type of malignant fibrous histiocytoma. After surgery, the patient was treated with adjuvant radiotherapy without systemic therapy. The tumor bed was treated with a computer tomography-based approach with a combination of three fields (left anterior oblique, right posterior oblique, and right lateral oblique) using an 18 MV linear accelerator (Figure 1).

**Figure 2:**
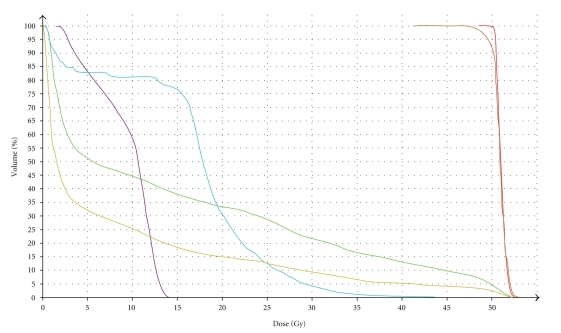
Dose-volume histogram: liver (green), left kidney (purple), body (pink), spinal cord (blue), CTV (red), and PTV (orange).

Treatment was given daily with a total dose of 50 Gy at 2.0 Gy per fraction, five days per week. Dose variation in the planning target volume (PTV) was kept within +7 and −5% of the prescribed dose according to ICRU recommendations [6, 7]. Radiation dose distribution was optimized to maximize target volume coverage while minimizing the dose to the organs at risk. Dose-volume histograms (DVHs) were recorded for the left kidney, liver, spinal cord, CTV, and PTV (Figure 2).

We adopted the Emami tolerance doses for the left kidney, which recommend TD5/5 of 50, 30, and 23 Gy for irradiation of one third of, two thirds of, or the entire kidney [8].

No acute or late toxicity and no postirradiation complications occurred, except for a G1 (RTOG toxicity criteria) acute cutaneous toxicity. The outcome shows that the patient remains alive twenty-four months after the surgery, without local recurrence or metastases.

## 3. Discussion

Malignant fibrous histiocytoma is a common soft-tissue sarcoma, usually occurring during the seventh decade of life. Fifty percent of cases originate in the lower limbs, 24% in the upper limbs, 16% in the trunk, and 9% in the retroperitoneum [2–5]. Primary renal MFH is a rare lesion characterized by symptoms common to other renal mass lesions. In the case reported here, preoperative differentiation from renal cell carcinoma was not feasible due to the atypical symptoms presented by the patient, such as abdominal pain and low-grade fever. The diagnostic imaging frequently includes CT and MRI but the definitive diagnosis is often a diagnosis of exclusion confirmed by histological analysis [9, 10], since immunohistochemistry allows a better definition of the histological characteristics of MFH. The case reported here was histologically identified as a fibroblastic-pleomorphic type of MFH. A renal biopsy was considered to identify the neoplasm as primary RCC. However, the clinical data indicated that a neoplasm and a nephrectomy would have been the curative choice in any case. Therefore, the renal biopsy was believed unnecessary. An inflammatory variety of MFH characterized by intense inflammatory infiltrate rich in neutrophils are also known, and although only a few cases of this type of MFH have been reported, it seems to be associated with a worse prognosis compared to fibroblastic-pleomorphic MFH [11, 12]. Although radical nephrectomy is currently the most beneficial treatment of MFH, adjuvant treatment is required to minimize the risk of local recurrence and distant metastases [9, 10, 13, 14]. A review of the literature suggests that cases of renal MFH have been reported describing clinicopathological [15–20] or imaging features [17–19] and surgery or chemotherapeutic treatment [9, 21, 22], but only minor data have been reported about radiotherapy [4, 10, 23, 24].

Different schedules of chemotherapy have been used for MFH, but due to the small number of cases, the conclusion regarding adjuvant chemotherapy is unclear [4, 9, 13, 18, 21, 22]. A similar scenario exists for the efficacy of the adjuvant radiotherapy on renal MFH. For the case presented in this study, no systemic therapy was administered and the patient was treated with radiotherapy alone. The maximum dose used for the left kidney was 14 Gy and the median dose was 11 Gy. Although the mean dose did not exceed tolerance, a total dose of 50 Gy was administered in order not to exceed the dose constraints. As previously reported, local recurrence and distant metastases are frequent in patients affected by renal MFH, leading to a poor prognosis for this type of tumor [11, 14, 15]. The case described by Singh and coworkers [11] was treated only with surgery and died within one month of diagnosis. Ishibiki and coworkers [14] described a case of storiform-pleomorphic malignant fibrous histiocytoma arising from perirenal tissue not treated with adjuvant therapy that developed local recurrence in the left retroperitoneal space after a followup period of 11 months. The unexpected outcome of the patient presented in this study seems to favor the use of adjuvant radiotherapy in the treatment of renal MFH. Although with a shorter followup time, a similar result has been showed by Eroylu and coworkers [23], which described a recurrence-free survival of fifteen months after surgery followed by radiotherapy with 6,600 rad. The efficacy of radiotherapy and its impact on this rare disease remains, however, unclear. It also has to be considered that significant technological improvements in radiotherapy have been achieved during the last years (e.g., multileaf collimators, electronic portal imaging, electronic administrative and image-data management systems, and computed tomography scanners) and that they might have improved also treatment results. The treatment of MFH has not been standardized yet and further studies would be necessary to improve the survival of patients and to better evaluate the most appropriate adjuvant treatment for this rare neoplasm.
